# A Systematic Review and Meta-Analysis of Risk Factors Associated with Severity and Death in COVID-19 Patients

**DOI:** 10.1155/2021/6660930

**Published:** 2021-04-10

**Authors:** Pengqiang Du, Dandan Li, Aifeng Wang, Su Shen, Zhichao Ma, Xingang Li

**Affiliations:** ^1^Department of Pharmacy, Fuwai Central China Cardiovascular Hospital, Henan Provincial People's Hospital, Central China Fuwai Hospital of Zhengzhou University, Zhengzhou, Henan 450003, China; ^2^Department of Pharmacy, Beijing Friendship Hospital, Capital Medical University, Beijing 100050, China; ^3^Department of Pharmacy, Affiliated Hospital of Chifeng University, Chifeng, Inner Mongolia 024000, China

## Abstract

This meta-analysis aims to screen the risk factors for severe illness and death and provide help for early clinical treatment of the new coronavirus (COVID-19). Based on a comprehensive search of PubMed, Embase, and Web of Science databases, we included studies that explored the cause and risk factors for severe illness and death in COVID-19 patients. We evaluated the strength of this relationship using odds ratios (ORs) with 95% confidence intervals (CIs). A total of 17 articles were included; 16 of the 17 articles were from China, and the risk factors associated with severe illness and death were age, sex, and multiple comorbidities. Advanced age (≥65 years, severe illness, OR = 2.62; death, OR = 6.00), male (severe illness, OR = 1.49; death, OR = 1.54), chronic respiratory diseases (severe illness, OR = 5.67; death, OR = 3.72), diabetes (severe illness, OR = 3.27; death, OR = 2.60), hypertension (severe illness, OR = 3.08; death, OR = 3.53), chronic kidney disease (severe illness, OR = 3.59; death, OR = 5.38), and cardiovascular diseases (severe illness, OR = 3.87; death, OR = 4.91) were all risk factors. For COVID-19 patients, advanced age, male, and patients with chronic disease are at higher risk of developing severe illness or even death.

## 1. Introduction

The novel coronavirus pneumonia (COVID-19) refers to pneumonia caused by the SARS-CoV-2 virus. COVID-19 is highly contagious, and it is mainly transmitted by respiratory droplets and close contacts. Prolonged exposure to a relatively closed high-concentration aerosol environment may spread the virus through aerosols [1]. As of October 22, 2020, the World Health Organization reported more than 41.3 million COVID-19 cases diagnosed globally, and the death toll exceeded 1,134,000. Although the majority of COVID-19 cases are classified as mild or moderate, the proportion of severe cases reached 14% with a death rate of 6.9% [2]. Severe patients generally present with dyspnea and hypoxemia. In critically ill cases, they can rapidly progress to acute respiratory distress syndrome (ARDS), septic shock, difficult-to-correct metabolic acidosis, coagulopathy, and multiple organ failure. Severely ill patients often require a variety of treatment modalities, such as antiviral drugs (interferon, lopinavir/ritonavir, chloroquine phosphate, etc.), ventilator-assisted treatment, and circulatory support. Severe patients have an extended length of hospital stay, even reaching 40 days, with an average cost of hundreds of thousands of dollars, a huge medical burden to the society.

In clinical practice, it is essential to screen risk factors for severe illness and death in COVID-19 patients and to detect potential or highly susceptible patients early. These initiatives can better allocate medical resources and promptly adjust treatment regimens to improve treatment efficacy and reduce the risk of death.

## 2. Materials and Methods

### 2.1. Protocol and Registration

This protocol follows the recommendations established by the Preferred Reporting Items for Systematic Reviews and Meta-Analyses (PRISMA) statement.

### 2.2. Literature Search

This meta-analysis is reported following the Preferred Reporting Items for Meta-Analyses (PRISMA) statement [3]. Two investigators (PD and DL) independently performed a systematic search in PubMed, Embase, and Web of Science databases with publication dates from December 1, 2019, to October 22, 2020. The following keywords were searched: (severe acute respiratory syndrome coronavirus 2 OR Wuhan seafood market pneumonia virus OR COVID-19 OR COVID19 OR coronavirus disease 2019 virus OR SARS-CoV-2 OR SARS2 OR 2019-nCoV OR 2019 novel coronavirus) AND (mortalities OR mortality OR fatality OR death OR acute respiratory distress syndrome OR ARDS OR ICU OR severe) AND risk factors. The retrieved citations were first assessed with title and abstract screening, and then full texts were checked based on the predefined inclusion and exclusion criteria.

### 2.3. Inclusion and Exclusion Criteria

Inclusion criteria were (1) studies on severe or death cases in COVID-19, (2) confirmed COVID-19 cases, (3) categorized severity types of COVID-19 based on standards, (4) human studies, (5) no limitation of types of studies, including research letters containing study data, and (6) no restriction in languages published. Exclusion criteria were (1) duplicate study, (2) study with incomplete data, (3) conference abstracts, reviews, and letters, (4) sample size less than three, and (5) studies with a poor-quality rating (the Newcastle–Ottawa Scale (NOS) < 7) or (Agency for Healthcare Research and Quality (AHRQ) < 8). Study selection was achieved by two investigators independently according to the inclusion and exclusion criteria. Any dispute was solved by discussion.

### 2.4. Quality Assessment

We assessed the quality of each included study using the following procedure. The quality of the selected studies was evaluated independently by two investigators (ZM and XL) according to a standardized set of criteria. Case-control or cohort studies were scored using the NOS score form, and the Quality in Prognosis Studies (QUIPS) tool [4] was used to evaluate the methodological quality of the literature full texts. A third reviewer (DL) was invited whenever there were disagreements.

### 2.5. Data Extraction

Data of the eligible studies were extracted in duplicate by two investigators (AW and SS) independently, and discrepancies were resolved by discussion with a third investigator (XL) or by consensus. From each publication, data on adjustment variables should be made as well.

### 2.6. Statistical Analysis

Our analysis was conducted using the Review Manager Software 5.3 (Cochrane Collaboration, Oxford, UK). ORs were used to describe risks between nonsevere and severe and deceased patients with regard to age, sex, and comorbidities. Random-effects models were used when there was a considerable heterogeneity (*P* < 0.1 or *I*^2^ > 50%) among studies. Otherwise, fixed-effects models were used. The *I*^2^-statistic and Cochran's *Q* test were used to assess statistical heterogeneity. The overall effects were calculated by a combined *Z*-value, with *P* < 0.5 indicating statistical significance.

### 2.7. Sensitivity Analysis and Publication Bias

Sensitivity analysis was performed by removing each study and calculating the *P* values of the remaining studies. Finally, publication biases were evaluated through funnel plot visual analysis.

## 3. Results

### 3.1. Study Identification

**Figure 1:**
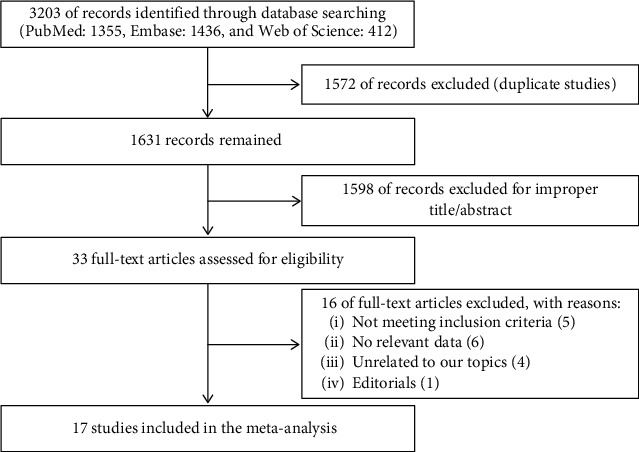
Flowchart of the identification of eligible trials.

A total of 3203 publications were retrieved based on the search criteria. After deleting duplicates, 1,631 articles were retained. Then, 1598 articles were excluded by reading the title and abstract. Of the remaining 33 articles, 16 were excluded due to not meeting inclusion criteria, not having relevant data, being unrelated to our topics, and editorials. And finally, 17 articles were included in this meta-analysis [1, 5–19]. The literature selection process is shown in Figure 1.

### 3.2. Study Characteristics

The base characteristics and quality assessment results are listed in Table 1, and the results of the risk of bias using the QUIPS tool are displayed in Table 2. All included publications had an NOS score of 7 or greater and an AHQR score of 8 or greater, and all were with low risk of bias. In the 17 articles included, all patients involved in the studies were infected by COVID-19, and the sample size ranged from 27 to 1,591, of which one was a two-way cohort study [9] and another one was a cross-sectional study [19]. The remaining 15 items were retrospective cohort studies. One study was from Italy and the other studies were from China, and the population was Asian and Italian. The details of the literature are listed in Supporting Material 1.

### 3.3. Statistical Analysis

#### 3.3.1. Advanced Age

**Figure 2:**
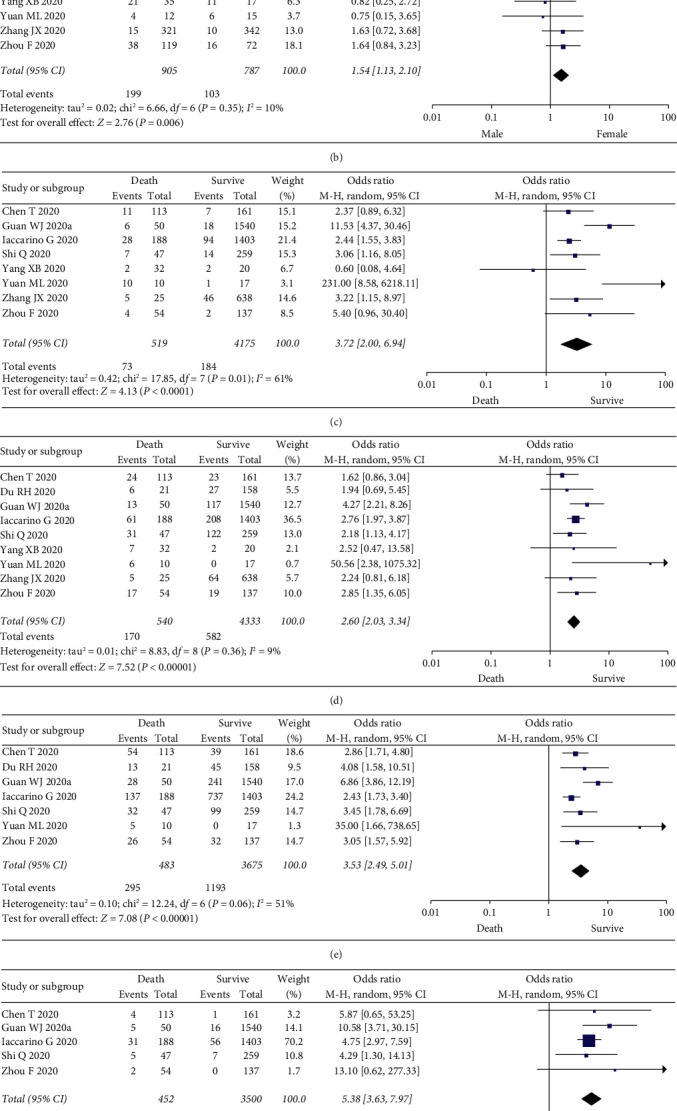
Meta-analysis results of the association between risk factors and death. (a) Advanced age vs. young age. (b) Male vs. female. (c) Chronic lung diseases. (d) Diabetes. (e) Hypertension. (f) Chronic kidney disease. (g) Cardiovascular disease.

**Figure 3:**
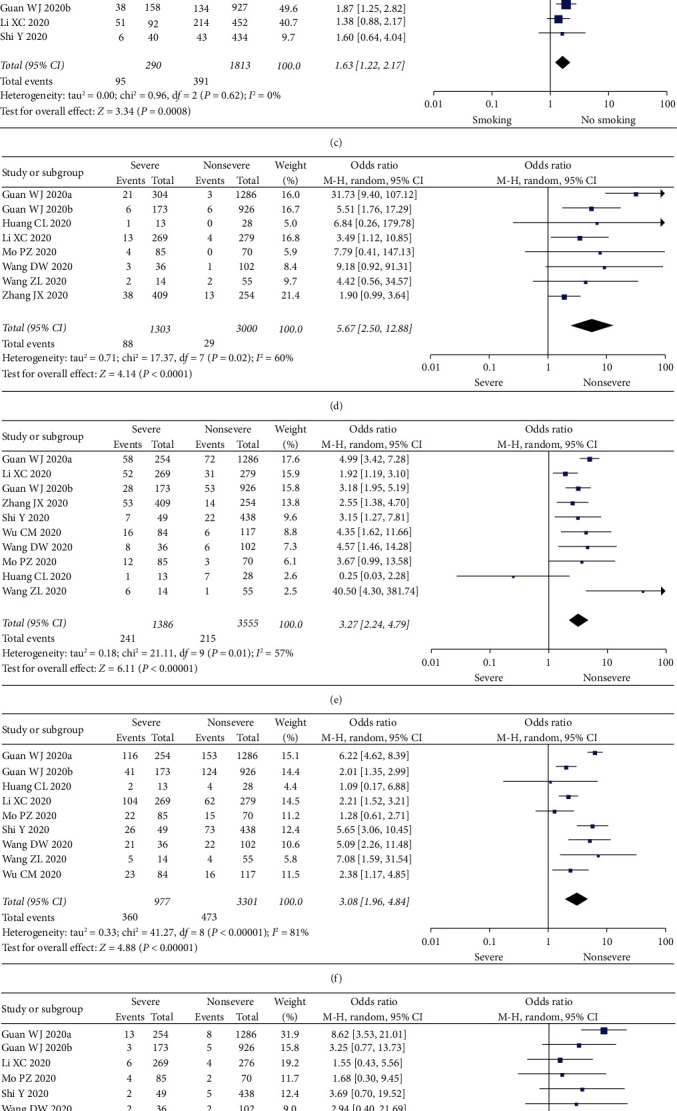
Meta-analysis results of the association between risk factors and severity. (a) Advanced age vs. young age. (b) Male vs. female. (c) Smoking vs. no smoking. (d) Chronic lung diseases. (e) Diabetes. (f) Hypertension. (g) Chronic kidney disease. (h) Cardiovascular disease.

There were 6 articles concerning the relationship between age and severity and death due to COVID-19. Three of the articles included patients older than 60 years old [5, 12], and the rest were older than 65 years old [1, 7, 9]. We separately analyzed the association between advanced age and severe illness and death. The results are shown in Figures 2(a) and 3(a). Older age was significantly associated with severe illness and death (severe illness, OR = 2.62, *I*^2^ = 0%, *n* = 2, and 95% CI = 2.01–3.42; death, OR = 6.00, *I*^2^ = 31%, *n* = 4, and 95% CI = 3.48–10.34).

#### 3.3.2. Sex

Two studies do not mention the association between sex and severe illness or death [6, 19]. All the included articles discussed the relationship between sex and the severity or death due to COVID-19. The results are shown in Figures 2(b) and 3(b). Meta-analysis results showed that male was significantly associated with severe illness and death (severe illness, OR = 1.49, *I*^2^ = 30%, *n* = 9, and 95% CI = 1.18–1.88; death, OR = 1.54, *I*^2^ = 10%, *n* = 7, and 95% CI = 1.13–2.10).

#### 3.3.3. Smoking

There were 4 articles mentioning the association between smoking and severe illness and death due to COVID-19, and 3 were included in the meta-analysis [1, 8, 9]. The remaining one [20] was excluded because it involved the association between the death due to COVID-19 and smoking. The results obtained are shown in Figure 3(c). Smoking was associated with severe illness (OR = 1.63, *I*^2^ = 0%, *n* = 3, and 95% CI = 1.22–2.17).

#### 3.3.4. Comorbidity

All included literature involved comorbidities, and we analyzed the relationship between comorbidities and the disease severity and the incidence of death. The results are shown in Figures 2(c)–2(g) and Figures 3(d)–3(h). Compared with the noncritically ill group, the proportions of patients with chronic respiratory diseases, diabetes, hypertension, chronic kidney disease, and cardiovascular system diseases in the severe and death groups were higher. All differences were statistically significant: chronic respiratory disease (severe illness, OR = 5.67, *I*^2^ = 60%, *n* = 8, and 95% CI = 2.50–12.88; death, OR = 3.72, *I*^2^ = 61%, *n* = 8, and 95% CI = 2.00–6.94); diabetes (severe illness, OR = 3.27, *I*^2^ = 57%, *n* = 10, and 95% CI = 2.24–4.79; death, OR = 2.60, *I*^2^ = 9%, *n* = 9, and 95% CI = 2.03–3.34); hypertension (severe illness, OR = 3.08, *I*^2^ = 81%, *n* = 9, and 95% CI = 1.96–4.84; death, OR = 3.53, *I*^2^ = 51%, *n* = 7, and 95% CI = 2.49–5.01); chronic kidney disease (severe illness, OR = 3.59, *I*^2^ = 19%, *n* = 6, and 95% CI = 1.90–6.76; death, OR = 5.38, *I*^2^ = 0%, *n* = 5, and 95% CI = 3.53–7.83); and cardiovascular diseases (severe illness, OR = 3.87, *I*^2^ = 0%, *n* = 10, and 95% CI = 2.99–5.01; death, OR = 4.91, *I*^2^ = 44%, *n* = 9, and 95% CI = 3.28–7.35).

#### 3.3.5. Sensitivity Analysis and Publication Bias

The sensitivity analysis was carried out by omitting one study at a time and calculating the *P* value of the remaining studies. The comparison results of COVID-19 severe illness and death among smokers and nonsmokers changed in the sensitivity analysis. There were only three studies included with a small sample size; therefore, the effect of smoking on the progression of COVID-19 needs to be further confirmed. The association of sex and COVID-19 death was not robust. Other findings of this meta-analysis were robust, and the results were not reversed by deleting individual studies.

**Figure 4:**
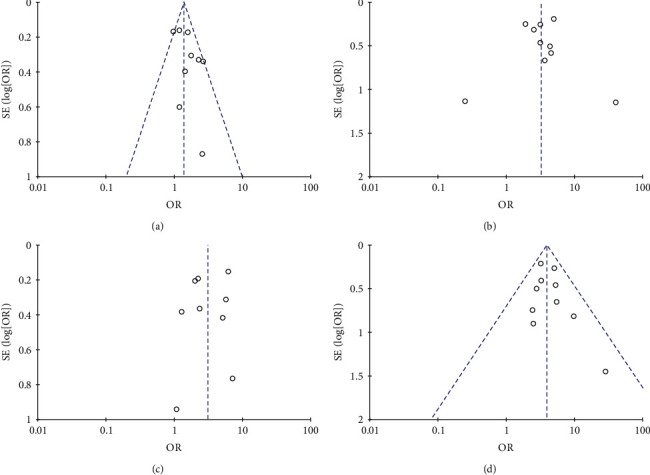
Funnel plots of meta-analysis including (a) sex and severe, (b) diabetes and severe, (c) hypertension and severe, and (d) cardiovascular disease and severe.

The publication bias was displayed visually using a funnel plot. Funnel plots were only used for four metagroups with relative more studies, including sex, diabetes, hypertension, and cardiovascular disease (Figure 4). The reported values of each literature were of symmetrical distribution on both sides of the overall OR value, indicating that there was no significant publication bias in the included studies.

## 4. Discussion

Our analysis shows that advanced age, males, and patient with chronic diseases (respiratory diseases, diabetes, hypertension, chronic kidney diseases, and cardiovascular diseases) are more likely to develop severe illness or death after being infected with the coronavirus. Smoking was associated with severe illness. However, after a sensitivity analysis, the conclusion was not stable. These results need to be verified by more studies with large sample sizes.

The coronavirus that causes COVID-19 belongs to the genus *β*-coronavirus. The spike protein (S protein) on the coat of the coronavirus plays a key role in the recognition of host cell receptors during virus infection [21]. For example, the S protein of SARS-CoV can bind to the angiotensin-converting enzyme 2 (ACE2) protein of alveolar epithelial cells and small intestinal epithelial cells, thereby mediating the virus to invade the cells to infect [22]. In addition to directly causing lung tissue damage, SARS-CoV-2 causes a cytokine storm that will further aggravate the inflammatory response. Abnormally elevated cytokines and overactivated immune cells are activated in the lung tissue, thereby causing diffuse damage to pulmonary capillary endothelial cells and alveolar epithelial cells. The accumulation of large amounts of exudate exacerbates the deterioration of lung function caused by airway obstruction, which aggravates ARDS and respiratory–circulatory failure. What is more serious is that it can develop into the uncontrolled systemic inflammatory response (SIRS), an essential factor leading to severe illness or death of COVID-19 patients [11, 23].

Statistics from countries around the world show that more than 70% of deaths are male patients, which means that the mortality rate of male patients with COVID-19 is almost 2.5 times that of females. Men are more likely to be infected with the coronavirus than women, probably because males have higher levels of ACE2 expression; for example, the ACE2 activity is 1.6-fold higher in the male kidneys [24, 25]. Another study analyzed the clinical data of thousands of patients with COVID-19. It showed that the coronavirus invaded the receptor of ACE2 in human cells, and the concentration in the blood of men was higher than that of women. In the index cohort, the mean plasma concentration was 5.38 in men compared with 5.09 in women (*P* < 0.001). In the validation cohort, the mean plasma concentration was 5.46 in men compared with 5.16 in women (*P* < 0.001). ACE2 is not only found in the lungs but also in the tissues of the heart, kidneys, and the lining of blood vessels. It is particularly high in the testes. This may partly explain why men have higher ACE2 concentrations, and therefore men are more likely to be infected with COVID-19 [26]. Besides, the stronger immune response of females may be due to more activated innate immune pathways prior to pathogen invasion. The protective effects of the female X chromosome and sex hormones play an important role in innate and adaptive immunity [27]. On the other hand, males may have higher unhealthy habits than women, such as a higher proportion of smoking and drinking. Our study also shows that men have a higher risk of COVID-19 infection than women (severe illness, OR = 1.49, *P*=0.0008; death, OR = 1.54, *P*=0.006). However, after a sensitivity analysis, the conclusion of the association of male and death was not stable. These results need to be verified by more studies with large sample sizes.

The body's immunity declines with age, and elderly patients are more likely to suffer from various dangerous diseases or death. In our included literature, the proportion of elderly patients involved in severe illness ranges from 4% to 37% and the percentage of elderly patients involved in deaths ranges from 10% to 38%. It can be seen that elderly patients account for a relatively high proportion of COVID-19 severe illness or death. The results of this study show that when patients are above 60 or 65 years of age, they are more likely to progress to severe disease or death when they are infected with COVID-19.

Comorbidities, such as chronic lung diseases, diabetes, hypertension, chronic kidney disease, and cardiovascular disease, may be linked to the pathogenesis of COVID-19. Chronic diseases share several standard features with infectious disorders, such as the proinflammatory state, and the attenuation of the innate immune response [28]. COVID-19 infection is caused by the binding of the virus surface spike protein to the ACE2 receptor. ACE2 is mainly present in the alveolar epithelium. Therefore, COVID-19 patients present with the most typical and significant lung involvement [29]. When a patient has a chronic respiratory disease, such as chronic obstructive pulmonary disease, the patient's lung function may be more severely damaged by the virus or even develop into ARDS [30]. Diabetes occurs in part because the accumulation of activated innate immune cells in metabolic tissues leads to the release of inflammatory mediators, especially IL-1*β* and TNF*α*, which promote systemic insulin resistance and *β*-cell damage [31]. SARS-CoV-2 may cause chronic inflammation, enhanced blood coagulation activity, and damage to the pancreas due to impaired immune response. This may be one of the potential mechanisms for the association between diabetes and COVID-19 [32]. Some studies have found that the prevalence of hypertension among patients infected with SARS-CoV-2 is about the same as the general population, or even lower [33]. However, some studies have mentioned that COVID-19 uses ACE2 to enter human cells, which may cause acute kidney injury and increase the risk of COVID-19 infection in patients with hypertension and diabetes [34, 35]. COVID-19 patients with cardiovascular diseases are more likely to have progressively weakened heart function due to other infectious diseases. When infected with SARS-CoV-2, these patients are more likely to have acute cardiovascular events and develop into severe infections. Acute heart injury and heart failure may be the main risk factors leading to death due to COVID-19 [5, 36]. Therefore, diseases such as chronic respiratory disease, diabetes, hypertension, chronic kidney disease, and cardiovascular disease are risk factors for severe illness and death of COVID-19 patients, which is consistent with the results of this meta-analysis.

A previous study reported that BMI was independently associated with the severity of COVID-19 in a multivariable analysis [37]. However, we cannot found this association due to the limited data. In our enrolled studies, the association between BMI and COVID-19 was not evaluated, and more research data were needed to investigate this association.

Our study has the following limitations: (1) the studies included are mostly from China. Racial differences may be present and may lead to bias in the results, (2) some studies have a small sample size, which may affect the reliability of the results, and (3) due to a large number of samples from Wuhan, some data may be duplicated.

In summary, we find through a meta-analysis that advanced age, male, and comorbidities of chronic respiratory disease, diabetes, hypertension, chronic kidney disease, and cardiovascular disease are risk factors for COVID-19 patients to develop severe illness or death. Clinicians should pay close attention to these risk factors and provide timely and personalized treatment modalities to enhance the efficacy and reduce the risk of death.

## Figures and Tables

**Table 1:** Characteristics and qualities of studies included in the meta-analysis.

PMID	Author	Year	Country	Ethnicity	Sample size	Mean age (year)	Male (%)	Type	NOS or AHQR
32217556	Chen T	2020	China	Chinese	274	62	62.4	Retrospective cohort	7
32217650	Guan WJ	2020	China	Chinese	1590	48.9 ± 16.3	56.8	Retrospective cohort	8
32269088	Du RH	2020	China	Chinese	179	57.6 ± 13.7	54.2	Retrospective cohort	8
32188484	Shi Y	2020	China	Chinese	487	46	53.2	Retrospective cohort	7
32294485	Li XC	2020	China	Chinese	548	60	50.9	Ambispective cohort	8
32304745	Zhang JX	2020	China	Chinese	663	55.6	48.4	Retrospective cohort	7
31986264	Huang CL	2020	China	Chinese	41	49	73.2	Retrospective cohort	7
32031570	Wang DW	2020	China	Chinese	138	56	54.3	Retrospective cohort	7
32105632	Yang XB	2020	China	Chinese	52	59.7	67.3	Retrospective cohort	7
32109013	Guan WJ	2020	China	Chinese	1099	47	58.1	Retrospective cohort	8
32167524	Wu CM	2020	China	Chinese	201	51	63.4	Retrospective cohort	7
32171076	Zhou F	2020	China	Chinese	191	56	62.3	Retrospective cohort	8
32191764	Yuan ML	2020	China	Chinese	27	56	44.4	Retrospective	7
32173725	Mo PZ	2020	China	Chinese	155	54	55.5	Retrospective	7
32176772	Wang ZL	2020	China	Chinese	69	42	46.4	Retrospective	7
32409504	Shi Q	2020	China	Chinese	306	65/64	49%	Retrospective	8
32564693	Iaccarino G	2020	Italy	Italian	1591	66.5 ± 0.4	64%	Cross-sectional	8

NOS: Newcastle–Ottawa Scale; AHQR: Agency for Healthcare Research and Quality.

**Table 2:** Risk of bias using QUIPS tool.

Study (PMID)	Study participation (max. 15)	Study attrition (max. 15)	Prognostic factor measurement (max. 15)	Outcome measurement (max. 15)	Statistical analysis and reporting (max. 15)	Quality score (max. 75)
32217556	15	12.5	12.5	15	15	70
32217650	15	12.5	15	15	15	72.5
32269088	15	12.5	12.5	15	15	70
32188484	15	10	12.5	15	15	67.5
32294485	15	12.5	15	15	15	72.5
32304745	15	10	12.5	15	15	67.5
31986264	15	10	12.5	15	15	67.5
32031570	15	10	12.5	15	15	67.5
32105632	15	10	12.5	15	15	67.5
32109013	15	10	12.5	15	15	67.5
32167524	15	10	12.5	15	15	67.5
32171076	15	12.5	12.5	15	15	70
32191764	15	10	10	15	12.5	62.5
32173725	15	12.5	12.5	15	15	70
32176772	15	10	12.5	15	15	67.5
32409504	15	10	12.5	15	15	67.5
32564693	15	12.5	10	15	15	67.5

QUIPS: quality in prognosis studies; the quality of the studies was ranked high if ≥60 points (≥80% of the maximum score); moderate if 45–59 points (≥60% and <80% of the maximum score); and low if <45 points (<60% of maximum score).

## Supplementary Materials

Supplementary Materials
*Supporting Material 1*. Characteristics of studies included in the meta-analysis.
