# Supplementary material for: A Systematic Review and Meta-Analysis of Risk Factors Associated with Severity and Death in COVID-19 Patients
Source: Can J Infect Dis Med Microbiol. 2021 Apr 10;2021:6660930. doi: 10.1155/2021/6660930 (PMC8040926; doi:10.1155/2021/6660930)
Supplement: Supplementary Materials — Supporting Material 1. Characteristics of studies included in the meta-analysis. [file 6660930.f1.pdf]

Supporting material 1. Characteristics of studies included in the meta-ana

PMID	AUTHOR	YEAR	COUNTRY	Ethnicity	SAMPLE SIZE
**32217556**	Chen T	2020	China	Chinese	274
**32217650**	Guan WJ	2020a	China	Chinese	1590
**32269088**	Du RH	2020	China	Chinese	179
**32188484**	Shi Y	2020	China	Chinese	487
**32294485**	Li XC	2020	China	Chinese	548
**32304745**	Zhang JX	2020	China	Chinese	663
**31986264**	Huang CL	2020	China	Chinese	41
**32031570**	Wang DW	2020	China	Chinese	138
**32105632**	Yang XB	2020	China	Chinese	52
**32109013**	Guan WJ	2020b	China	Chinese	1099
**32167524**	Wu CM	2020	China	Chinese	201
**32171076**	Zhou F	2020	China	Chinese	191
**32191764**	Yuan ML	2020	China	Chinese	27

**32173725**	Mo PZ	2020	China	Chinese	155
**32176772**	Wang ZL	2020	China	Chinese	69
**32409504**	Shi Q	2020	China	Chinese	306
**32564693**	Iaccarino G	2020	Italy	Italian	1591

lysis

CASE CHARACTERISTICS
mean age 62 years; 171 males, 103 females; <40 years 53, 40-60 years 68, ≥60years 153
mean age: 48.9±16.3 years; 904 males; 674 females
mean age 57.6±13.7 years; 97 males, 82 females; 0-49 years 49, 50-64 years 65; ≥65 years 65
mean age 46 years; 259 males, 228 females
mean age 60 years; 279 males, 269 females; 0-44 years 107, 45-64 years 231, ≥65 years 210
mean age 55.6 years; 321 males, 342 females; 0-60 years 348, > 60 years 315
mean age 49 years; 30 males, 11 females
mean age 56 years; 75 males, 63 females
mean age 59.7 years; 35 males, 17 females; 30-60 years 25, ＞60 years 27
mean age: 47 years; 639males; 460 females; 0-65 years 849; ≥65 years 153
mean age 51 years; 128 males, 73 females
mean age 56 years; 119 males, 72 females
mean age 56 years; 12 males, 15 females

mean age 54 years; 86 males, 69 females
mean age 42 years; 32 males, 37 females
Patients without diabetes: mean age 65 years; Patients with diabetes: mean age 64 years; each group: 75 males, 78 females
mean age 66.5±0.4 years; 1018 males, 573 females

Study design
A retrospective study obtained epidemiological, clinical, laboratory, and radiological characteristics, as well as treatment and outcome data, from electronic medical records for deceased patients and recovered patients by using data collection forms.
A retrospective case study compiled the clinical data of laboratory-confirmed hospitalized cases from 575 hospitals between December 11th, 2019 and January 31st, 2020
The written informed consent from each patient was waived since we prospectively collected and analyzed all data from each patient according to the policy for public health outbreak investigation of emerging infectious diseases issued by the National Health Commission of the People's Republic of China.
A retrospective study obtained medical records, laboratory findings, and pulmonary CT scan of each patient with COVID-19, provided by the local health authority and inputted into a pre-specified electronic data collection form.
The epidemiological and demographic data were obtained by face-to-face or telephone interview. Clinical symptoms, laboratory, and radiological findings on admission as well as the complications, treatment and outcomes during hospitalization were extracted from electronic medical records.
A retrospective study obtained oral informed consent from all patients enrolled in the study.
Local centres for disease control and prevention collected respiratory, blood, and faeces specimens, then shipped them to designated authoritative laboratories to detect the pathogen.
The datawere reviewed by a trained team of physicians. Information recorded included demographic data, medical history, exposure history, underlyingcomorbidities, symptoms,signs,laboratoryfindings,chest computed tomographic (CT) scans, and treatment measures (ie, antiviral therapy, corticosteroid therapy, respiratory support, kidney replacement therapy).
The retrospective, observational study obtained clinical electronic medical records, nursing records, laboratory findings, and radiological examinations for all patients with laboratory confirmed SARS-CoV-2 infection.
The study obtained the medical records and compiled data for hospitalized patients and outpatients with laboratory-confirmed Covid-19.
A trained team of physicians and medical students reviewed andcollectedepidemiological, clinical,andoutcomesdata from electronic medical records.
Epidemiological, demographic, clinical, laboratory, treatment, and outcome data were extracted from electronic medical records using a standardised data collection form.
Clinical characteristics together with chest imaging manifestations of each confirmed cases were recorded.

A COVID-19 case report form was designed to document primary data regarding demographic, clinical, laboratory, radiological and therapeutic characteristics from electronic medical records
The study obtaines clinical charts, nursing records, laboratory results, and chest CT characteristics for all patients.
Epidemiological, demographic, clinical, laboratory, treatment,<br>and outcome data were extracted from electronic medical records using a standardised data collection form.
An online questionnaire was distributed among the centers to collect reviewed epidemiological, clinical, and outcomes data from hospital emergency rooms and regular and intensive care wards.

Clinical typing of COVID-19(No)	Young age(<60 or <65 years)	advanced age	Male
Recovered patients: 161; Deaths: 113	Recovered patients: 102; Deaths: 19	Recovered patients: 59; Deaths: 94	Recovered patients: 88; Deaths: 83
Mild: 1286; Severe: 254; Deaths: 50	-	-	-
Deceased: 21; Survivors: 158	Survivors: 110; Deceased: 4	Survivors: 48; Deceased: 17	Survivors: 87; Deceased: 10
Mild: 438; Severe: 49	-	-	Mild: 223; Severe: 36
Nonsevere: 279; Severe: 269	Nonsevere: 204; Severe: 134	Nonsevere: 75; Severe: 135	Nonsevere: 126; Severe: 153
Mild to Moderate: 254; Severe: 315; Critical: 94; Survival: 638, Non-survi val: 25	Mild to Moderate: 185; Severe: 136; Critical: 27; Survival: 342, Non-survi val: 6	Mild to Moderate: 69; Severe: 179; Critical: 67; Survival: 296, Non-survival: 19	Mild to Moderate: 116; Severe: 149; Critical: 56; Survival: 306, Non-survi val: 15
Mild: 28; Severe: 13	-	-	Mild: 19; Severe: 11
Mild: 102; Severe: 36	-	-	Mild: 53; Severe: 22
Deceased: 32; Survivors: 20	Survivors: 13; Deceased: 12	Survivors: 7; Deceased: 20	Survivors: 14; Deceased: 21
Mild: 926; Severe: 173	Mild: 739; Severe: 119	Mild: 109; Severe: 44	Mild: 537; Severe: 100
Mild: 117; Severe: 84	-	-	Mild: 68; Severe: 60
Deceased: 54; Survivors: 137	-	-	Survivors: 81; Deceased: 38
Survivors: 17; Deceased: 10	-	-	Survivors: 8; Deceased: 4

Mild: 70; Severe: 85	-	-	Mild: 31; Severe: 55
Mild: 55; Severe: 14	-	-	Mild: 15; Severe: 7
Survival: 259; Death: 47	-	-	Survivors: 122; Death: 28
Nonsurvivors: 188; Survivors: 1403	-	-	-

Female	Smoking	No Smoking	Chronic lung diseases
Recovered patients: 73; Deaths: 30	Recovered patients: 10; Deaths: 9	Recovered patients: 151; Deaths: 104	Recovered patients: 7; Deaths: 11
-	-	-	Mild: 3; Severe: 15; Deaths: 6
Survivors: 71; Deceased: 11	-	-	-
Mild: 215; Severe: 13	Mild: 34; Severe: 6	Mild: 391; Severe: 43	-
Nonsevere: 153; Severe: 116	Nonsevere: 41; Severe: 51	Nonsevere: 238; Severe: 214	Nonsevere: 4; Severe: 13
Mild to Moderate: 138; Severe: 166; Critical: 38; Survival: 332, Non-survi val: 10	-	-	Mild to Moderate: 13; Severe: 23; Critical: 15; Survival: 46, Non-survi val: 5
Mild: 9; Severe: 2	-	-	Mild: 0; Severe: 1
Mild: 51; Severe: 14	-	-	Mild: 1; Severe: 3
Survivors: 6; Deceased: 11	-	-	Survivors: 2; Deceased: 2
Mild: 386; Severe: 73	Mild: 120; Severe: 38	Mild: 793; Severe: 134	Mild: 6; Severe: 6
Mild: 49; Severe: 24	-	-	-
Survivors: 56; Deceased: 16	-	-	Survivors: 2; Deceased: 4
Survivors: 9; Deceased: 6	-	-	Survivors: 1; Deceased: 10

Mild: 39; Severe: 30	-	-	Mild: 0; Severe: 4
Mild: 30; Severe: 7	-	-	Mild: 2; Severe: 2
Survivors: 137; Death: 19	-	-	Survivors: 14; Death: 7
-	-	-	Nonsurvivors: 28; Survivors: 94

Diabetes	Hypertension	Chronic kidney disease	Cardiovascular disease	TYPE
Recovered patients: 23; Deaths: 24	Recovered patients: 39; Deaths: 54	Recovered patients: 1; Deaths: 4	Recovered patients: 7; Deaths: 16	retrospective cohort
Mild: 72; Severe: 45; Deaths: 13	Mild: 153; Severe: 88; Deaths: 28	Mild: 8; Severe: 8; Deaths: 5	Mild: 31; Severe: 20; Deaths: 8	retrospective cohort
Survivors: 27; Deceased: 6	Survivors: 45; Deceased: 13	-	Survivors: 17; Deceased: 12	retrospective cohort
Mild: 22; Severe: 7	Mild: 73; Severe: 26	Mild: 5; Severe: 2	Mild: 7; Severe: 4	retrospective cohort
Nonsevere: 31; Severe: 52	Nonsevere: 62; Severe: 104	Nonsevere: 4; Severe: 6	Nonsevere: 6; Severe: 28	ambispective cohort
Mild to Moderate: 14; Severe: 39; Critical: 14; Survival: 64, Non-survival: *5*	-	-	Mild to Moderate: 33; Severe: 82; Critical: 49; Survival: 148, Non-survival: 16	retrospective cohort
Mild: 7; Severe: 1	Mild: 4; Severe: 2	-	Mild: 3; Severe: 3	retrospective cohort
Mild: 6; Severe: 8	Mild: 22; Severe: 21	Mild: 2; Severe: 2	Mild: 11; Severe: 9	retrospective cohort
Survivors: 2; Deceased: 7	-	-	Survivors: 2; Deceased: 3	retrospective cohort
Mild: 53; Severe: 28	Mild: 124; Severe: 41	Mild: 5; Severe: 3	Mild: 17; Severe: 10	retrospective cohort
Mild: 6; Severe: 16	Mild: 16; Severe: 23	-	Mild: 3; Severe: 5	retrospective cohort
Survivors: 19; Deceased: 17	Survivors: 32; Deceased: 26	Survivors: 0; Deceased: 2	Survivors: 2; Deceased: 13	retrospective cohort
Survivors: 0; Deceased: 6	Survivors: 0; Deceased: 5	-	Survivors: 0; Deceased: 3	retrospective

Mild: 3; Severe: 12	Mild: 15; Severe: 22	Mild: 2; Severe: 4	Mild: 0; Severe: 14	retrospective
Mild: 1; Severe: 6	Mild: 4; Severe: 5	-	Mild: 3; Severe: 5	retrospective
Survivors: 122; Death: 31	Survivors: 99; Death: 32	Survivors: 7; Death: 5	Survivors: 32; Death: 17	retrospective
Nonsurvivors: 61; Survivors: 208	Nonsurvivors: 137; Survivors: 737	Nonsurvivors: 31; Survivors: 56	Nonsurvivors: 56; Survivors: 160	cross-sectional

Studies quality (AHQR or NOS)
7
8
8
7
8
7
7
7
7
8
7
8
7

7

7

8

8
